# Nanoscale myelinogenesis image in developing brain via super-resolution nanoscopy by near-infrared emissive curcumin-BODIPY derivatives

**DOI:** 10.1186/s12951-024-02377-9

**Published:** 2024-03-11

**Authors:** Junyang Chen, Yifan Yu, Siyou Wang, Yu Shen, Yupeng Tian, Loris Rizzello, Kui Luo, Xiaohe Tian, Tinghua Wang, Liulin Xiong

**Affiliations:** 1 Department of Anesthesiology, Affiliated Hospital of Zunyi Medical University, No. 149, Dalian Road, Huichuan District, Zunyi, 563000 Guizhou China; 2 Functional and Molecular Imaging Key Laboratory of Sichuan Province, Huaxi MR Research Centre (HMRRC), Department of Radiology and National Clinical Research Center for Geriatrics, West China Hospital of Sichuan University, Chengdu, 610000 China; 3 Institute of Neurological Disease, Translational Neuroscience Center, West China Hospital, Sichuan University, Chengdu, 610041 China; 4 Department of Chemistry, Key Laboratory of Functional Inorganic Material Chemistry of Anhui Province, Anhui University, Hefei, 230601 China; 5 Department of Pharmaceutical Sciences, University of Milan, Via G. Balzaretti 9, 20133 Milan, Italy; 6 The National Institute of Molecular Genetics (INGM), Via Francesco Sforza 35, 20122 Milan, Italy; 7 Department of Chemistry, University College London, London, WC1H 0AJ UK; 8 Laboratory of Aging Research and Cancer Drug Target, State Key Laboratory of Biotherapy and Cancer Center, West China Hospital, Sichuan University, Chengdu, 610041 China

**Keywords:** Nanoscale myelinogenesis image, Super-resolution, Developing Brain, Near-Infrared probe

## Abstract

Understanding the intricate nanoscale architecture of neuronal myelin during central nervous system development is of utmost importance. However, current visualization methods heavily rely on electron microscopy or indirect fluorescent method, lacking direct and real-time imaging capabilities. Here, we introduce a breakthrough near-infrared emissive curcumin-BODIPY derivative (**MyL-1**) that enables direct visualization of myelin structure in brain tissues. The remarkable compatibility of **MyL-1** with stimulated emission depletion nanoscopy allows for unprecedented super-resolution imaging of myelin ultrastructure. Through this innovative approach, we comprehensively characterize the nanoscale myelinogenesis in three dimensions over the course of brain development, spanning from infancy to adulthood in mouse models. Moreover, we investigate the correlation between myelin substances and Myelin Basic Protein (MBP), shedding light on the essential role of MBP in facilitating myelinogenesis during vertebral development. This novel material, **MyL-1**, opens up new avenues for studying and understanding the intricate process of myelinogenesis in a direct and non-invasive manner, paving the way for further advancements in the field of nanoscale neuroimaging.

**Graphical Abstract:**

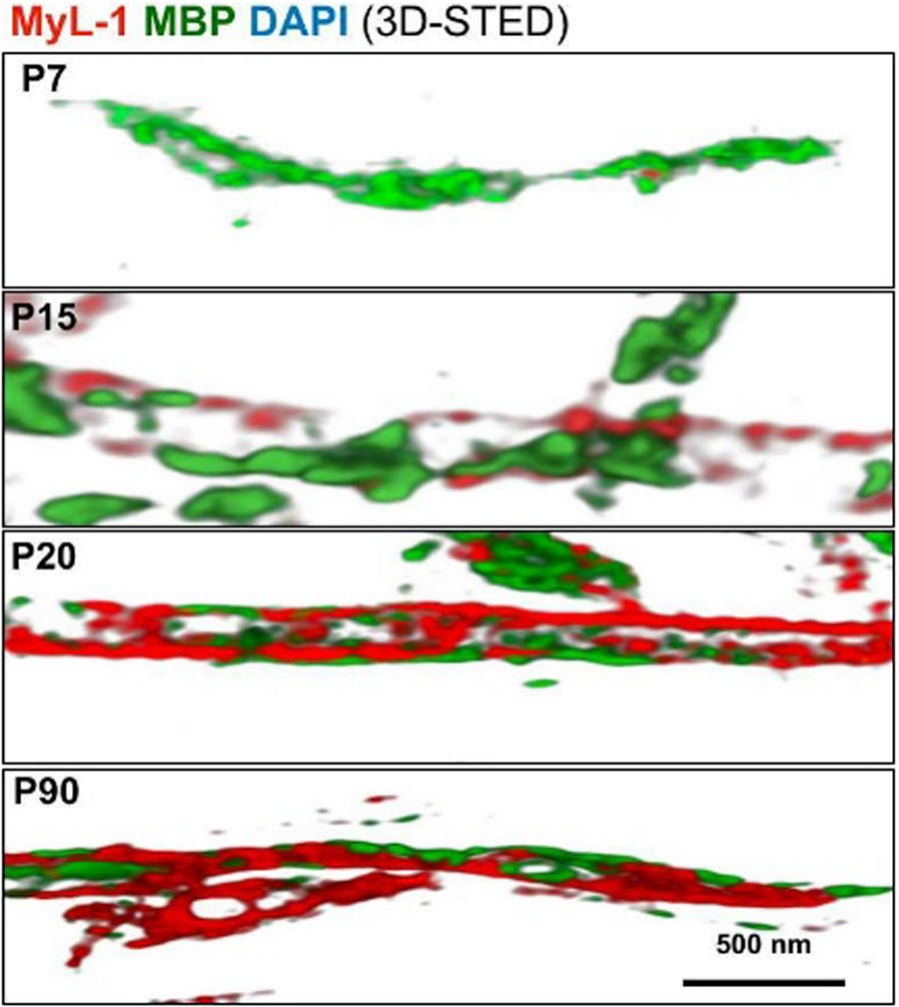

**Supplementary Information:**

The online version contains supplementary material available at 10.1186/s12951-024-02377-9.

## Introduction

Neuronal signaling transduction is critical for all the physiological functions of the nervous system in both healthy and disease-related conditions [1–3]. Mammal nervous system integrity is necessary to achieve a correct information transfer between and within neurons. The process is heavily dependent on myelinogenesis, namely the formation of lipid-rich substances to insulate the neuron axon [4–6]. It is believed that the evolution of cognitive (e.g. language) and motor skills (e.g. walking) is closely related to successful myelin formation and development from infancy to maturity [2, 7, 8]. The loss of myelin sheath (also called demyelination) can directly cause a rapid decrease in neuronal electrical impulse speed. This is the hallmark of several neurodegenerative autoimmune (e.g., multiple sclerosis) and inherited demyelinating diseases (e.g. leukodystrophy) [9–11]. Understanding myelin ultrastructures, and in particular its dynamics during development, remain indeed of great significance for neuroscience.

Electron microscopy (EM) is traditionally the most commonly used technique to observe myelin structure, as it allows reaching nanoscale resolution [12]. An obvious disadvantage of EM is the difficulty in measuring large scale images quantitatively, as well as 3-dimensional (3D) or time-lapse information would require a heavy and complex workload [13]. Also, samples for EM might risk losing ultrastructure during the hash preparation process (e.g. oxidation and dehydration) [14]. Visualizing myelin structures by organic probes-based fluorescent microscopy can be a good alternative since the cells could retain their natural aqueous environment [15]. Moreover, the recent advancements in the field of super-resolution nanoscopy, e.g., stimulated emission depletion (STED), Structure Illumination Microscopy (SIM) and stochastic optical reconstruction microscopy (STROM), make it feasible to access myelin ultrastructure also at in vivo level [16]. However, previous studies typically use an indirect method by staining MBP [17, 18]. Nevertheless, commercialized probes for myelin structure are extremely rare and none of these were found to be suitable for super-resolution nanoscopy with exquisite sensitivity. An ideal myelin labeling probe should combine high specificity against sphingomyelin in parenchymal tissue together with high resilience towards intense laser irradiation (typical of STED), especially in the perspective of reconstructing 3D ultrastructure in super resolution.

Herein, we report a novel dibranched curcumin-BODIPY derivative **MyL-1** with an emission in the near-infrared region I (NIR-I). We found **MyL-1** can specifically mark neuronal myelin allowing observation of 3D ultrastructure during myelinogenesis under STED nanoscopy. The current work reveals new insights in the dynamic variations of myelin structure at both tissue- and single-cell level, and a correlation with MBP is revealed. These results offered valuable information to understand myelinogenesis during vertebrate development and related neurodegenerative disease.

## Results and discussion

### Design strategies and photophysical properties of MyL compounds

**Fig. 1:**
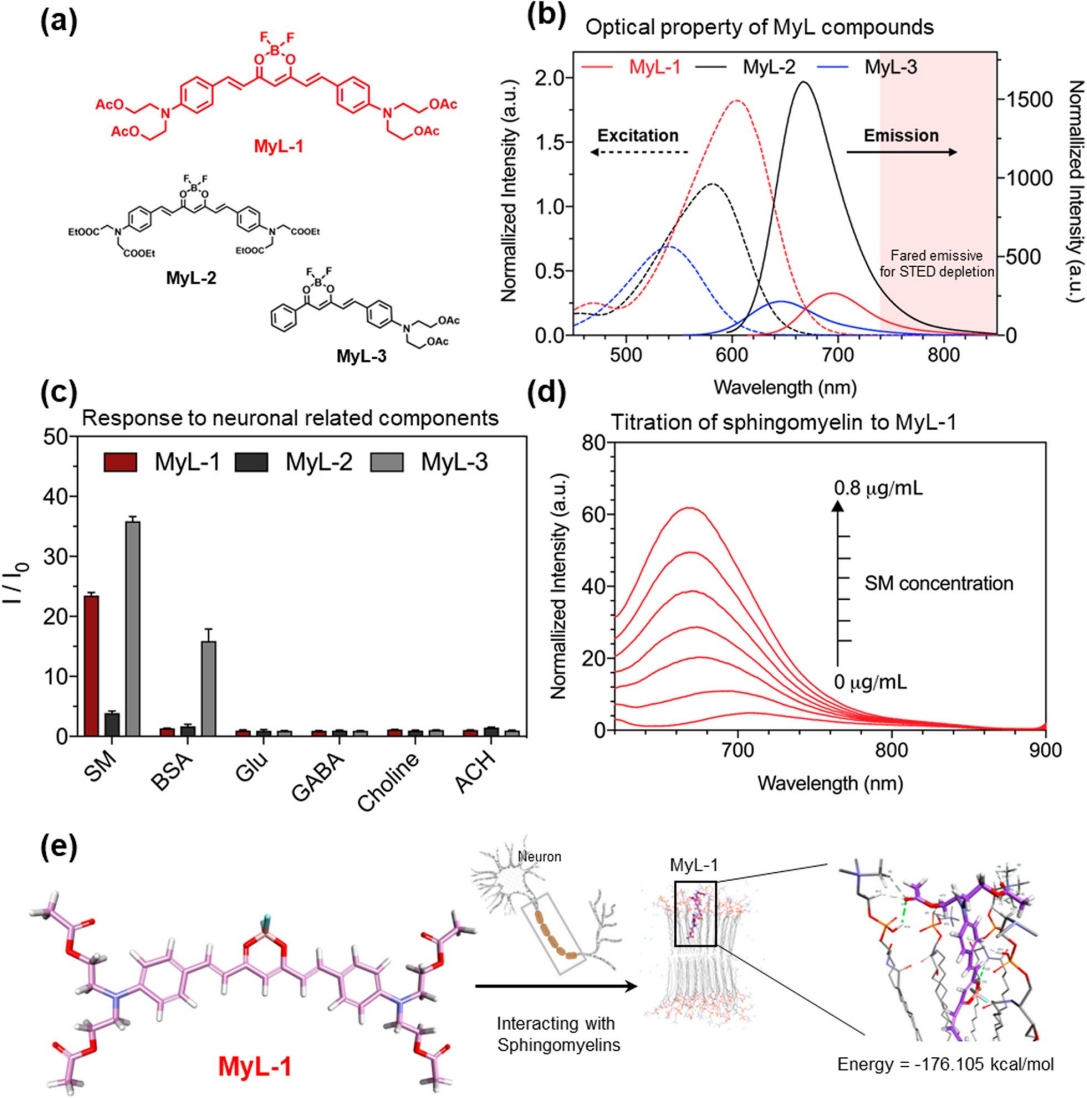
**a** Chemical structure of **MyL-1**, **MyL-2** and **MyL-3**. **b** UV–vis absorption and Fluorescence spectra of **MyL-1**, **MyL-2** and **MyL-3** in DMSO solution (Concentration = 10 mM). **c** Fluorescence response analysis of **MyL-1**, **MyL-2** and **MyL-3** (5 μM) against Sphingomyelins (SM, 10 mg/ml), Bovine Serum Albumin (BSA, 30 g/ml), glutamic acid (Glu, 10 mM), γ-aminobutyric acid (GAGB, 10 mM), Choline chloride (Choline, 10 mM), Acetylcholine chloride (ACH, 10 mM) (I: intensity, I_0_: solvent intensity). **d** Fluorescence titration experiments of **MyL-1** (5 μM) with sphingomyelin showed emission enhancement. **e** The binding energy for the interaction of **MyL-1** molecule with sphingomyelins aggregates as calculated by molecular dynamics calculations

Here, an O-B-O motif BODIPY was introduced to construct three molecular probes named **MyL-1**, **MyL-2** and **MyL-3** (Fig. 1a), the N atom in a typical N-B-N-based BODIPY-derivative molecule was completely replaced by more electron-rich O atom to obtain an O-B-O-type BODIPY derivative molecules, resulting in excellent photophysical properties [19, 20]. The introduction of flexible ester groups at the end of the molecules (**MyL-1** and **MyL-3**) leads better biocompatibility [21] and might favour targeting lipid-rich myelin substances. Also, the ester group with different push–pull electronic effects facilitates the regulation of the optical properties of the compound [22].

The three compounds **MyL-1**, **MyL-2,** and **MyL-3** were synthesized based on the above strategies (detailed synthesis refer to Additional file 1: and Schemes S1–S3) and characterized by ^1^H/^13^C and NMR spectroscopy (Additional file 1: Figs. S1–S6). Their photophysical properties, including excitation and emission, were investigated (Fig. 1b and Additional file 1: Table S1), and strengthened by theoretical calculation used elsewhere (Additional file 1: Fig. S7) [23]. All the compounds possess a strong red-emission, starting at 600 nm onwards. In particular, the **MyL-1** showed an emission peak at 700 nm, with a relatively bigger stock shift (~ 100 nm), which is favorable for STED evaluation (STED depletion = 775 nm) to avoid the probe excited by depletion power [24]. Its red-emissive and large stock-shift is highly due to the terminal di-branched ester group that can enlarge the conjugated system of the molecule compared to the single branch system. The resulting intramolecular charge transfer (ICT) process is thus significantly increased in the whole molecular system.

### In vitro selectivity assessment of MyL compound against sphingomyelin

After successfully obtaining the **MyL-1**, **MyL-2,** and **MyL-3** compounds with excellent photophysical properties, their sensitivity and selectivity against myelin substances were assessed by fluorescence response experiments. Sphingomyelin (SM) has been chosen as the main component of myelin sheath. Conversely, control experimental groups included bovine serum albumin BSA, glutamic acid (Glu), γ-aminobutyric acid (GABA), choline chloride (Choline), and acetylcholine chloride (ACH). Interestingly, both **MyL-1** and **MyL-3** displayed a noticeable emission enhancement, suggesting that these two compounds could specifically bind SM (Fig. 1c). It is noteworthy that although **MyL-3** displayed stronger enhancement than **MyL-1**, its unspecific reaction with protein (BSA) hindered further applications at ex vivo level. Titration experiments further confirmed the specificity and selectivity of **MyL-1** against SM (Fig. 1d), as an increase in concentration from 0 to 0.8 μg/mL led to a detected ~ 16 folds fluorescence enhancement for the **MyL-1** compound.

### Bilayer setup and simulation

As different lipids lead to different structural properties of the bilayer, which affect biological function, the SM bilayer was modeled to understand the impact of the presence of **MyL-1**, **MyL-2,** and **MyL-3** molecules. In this section, SM lipids are monounsaturated, having unequal sn-1 and sn-2 chains, i.e. 16:1 and 18:0 fatty acids as hydrophobic tails. The structure of the lipids is shown in Fig. 1e. The bilayer model applies the CHARMM force field and rescues a binary system of a total of 64 lipids (32 per leaf) in a rectangular box with a hydration number of 95 water molecules per lipid. MD simulations were performed using a standard dynamic cascade protocol. The system was energy minimized using a 1000-step steepest descent algorithm, followed by a five-step equilibrium run in which an NP (pressure) T set consisting of a constant number of atoms, pressure, and temperature was performed. Production runs of at least 10 ns were used in the NPT ensemble at 300 K and 1 bar. The total energy of interaction of molecules **MyL-1**, **MyL-2,** and **MyL-3** (Additional file 1: Fig. S8) with sphingolipids was − 176.105441 (kcal/mol), − 125.983062 (kcal/mol) and − 111.54 (kcal/mol), respectively, indicating that **MyL-1** interacted most strongly with sphingolipids.

**Fig. 2:**
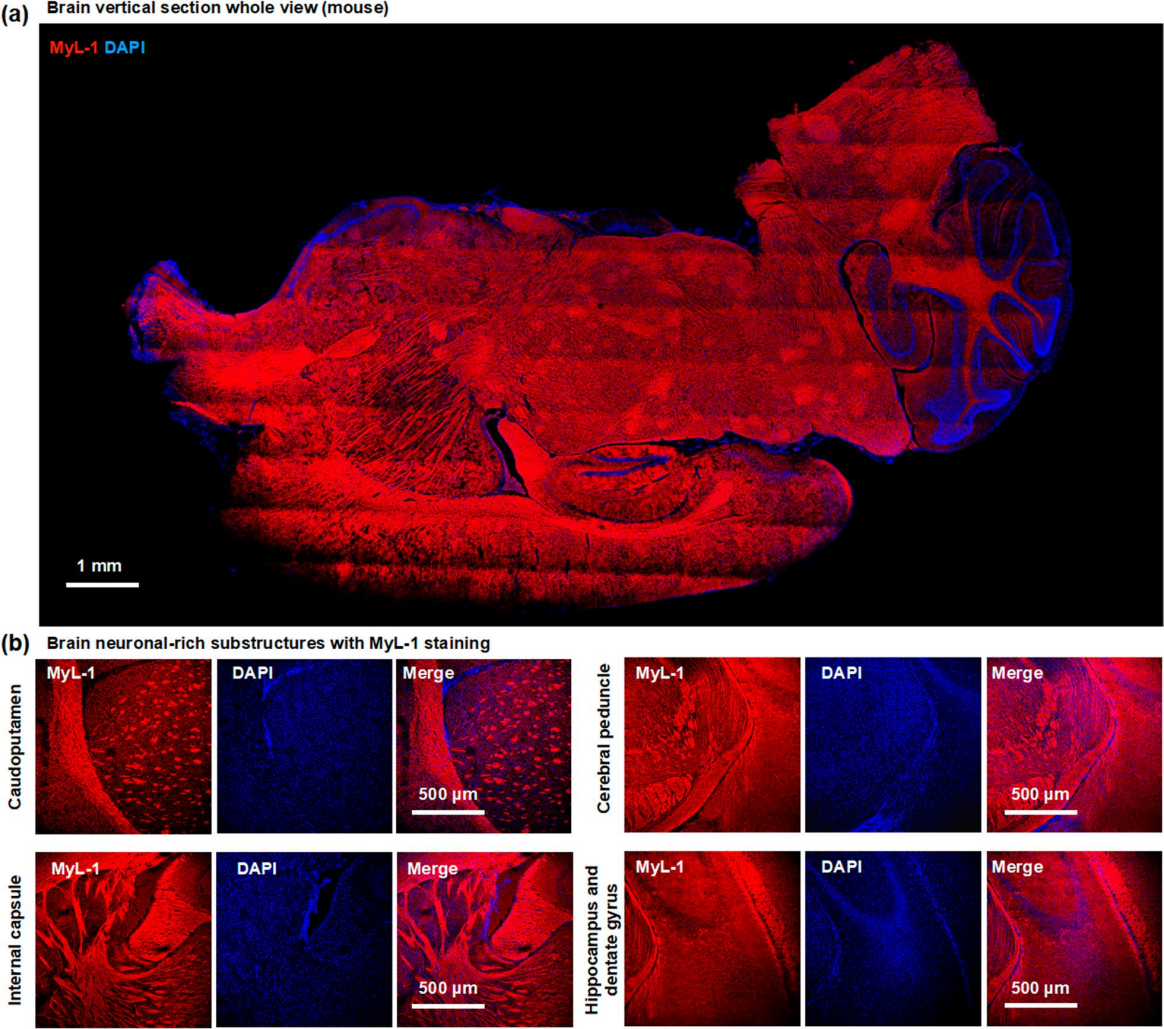
**a** Confocal images of whole fresh mouse brain vertical section incubated with **MyL-1** compound (20 μM) for 30 min, and cell nuclear was highlighted by DAPI. **b** Higher magnification micrographs from axon rich regions including caudoputamen, cerebral peduncle, internal capsule, hippocampus, and dentate gyrus

The significant fluorescence enhancement of **MyL-1** with sphingomyelin provides the possibility to visualize myelin sheath in vivo. The relatively low cytotoxicity of **MyL-1** also offered the possibility to explore its application on live tissues (Additional file 1: Fig. S9). Fresh mouse brain sections were subsequently incubated with **MyL-1** (20 μM) and imaged directly without fixation under laser scanning microscopy. **MyL-1** signal (Fig. 2a) could be detected throughout the whole brain vertical section with clear fibril staining pattern. Further neuronal axon-rich regions were highlighted (Fig. 2b), and included caudoputamen, cerebral peduncle, internal capsule, hippocampus, and dentate gyrus, suggesting **MyL-1** signal might correspond to the myelin sheaths of axonal protrusions.

### Colocalization assessment and myelin targeting of MyL-1 in brain tissue

**Fig. 3:**
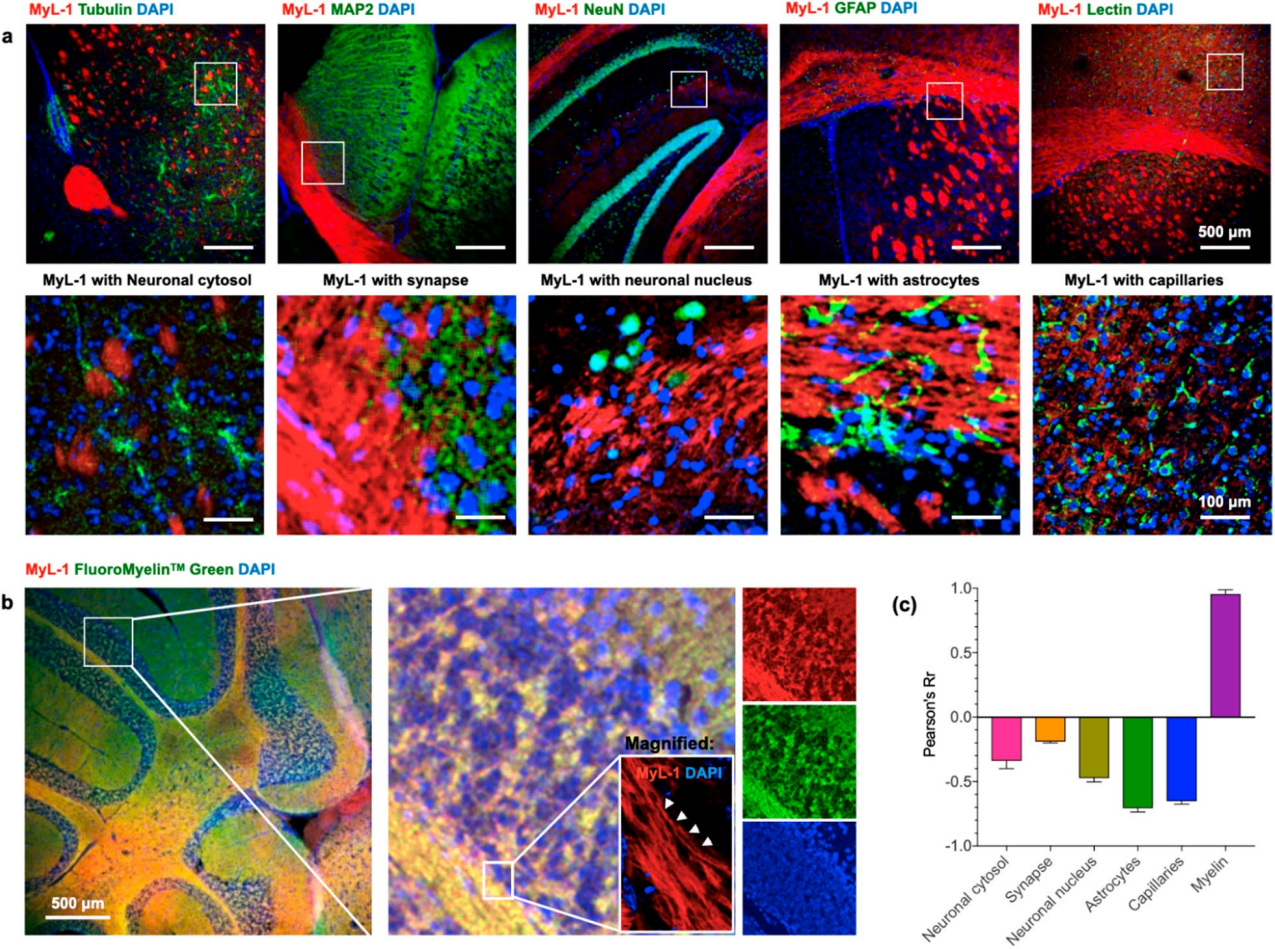
**a** Confocal images of fresh mouse brain section incubated with **MyL-1** compound (20 μM) for 30 min, the co-stained or immunostained with other brain parenchymal marker, including tubulin for neuronal cytosol, MAP2 for synapse, NeuN for neuronal nucleus, GFAP for astrocytes and Lectin for brain capillaries, lower row are magnified micrographs from selected region from upper row. Cell nucleus were marked by DAPI. **b** Confocal images of fresh mouse brain section incubated with **MyL-1** compound (20 μM) for 30 min, then co-stained with FluoroMyelinTM Green, magnified images showed a high overlap degree. **c** Colocalization analysis indicated by Pearson Coefficiency Rr from the above co-stain experiments (n = 10–20 images from three independent experiments)

To confirm that **MyL-1** could specifically light up only neuronal myelin components, without disturbing other numerous parenchymal substances, a detailed colocalization experiment was performed. The brain slices were firstly incubated with **MyL-1** for 30 min and subsequently immunofluorescently (or fluorescently) labeled to highlight neuronal cytoskeleton (anti-beta-tubulin), neuronal synapse (anti-MAP2, microtubule-associated protein 2), neuronal nucleus (anti-NeuN, neuronal nuclear associate protein), astrocytes (anti-GFAP, Glial fibrillary acidic protein) and brain capillary endothelium (FITC-labelled Lectin). The results indicated hardly any overlapping signal between **MyL-1** and the other substances listed above (Fig. 3a and relative magnified micrograph). In a good contrast, **MyL-1** demonstrated a strong colocalization with FluoroMyelin™ Green (FMG) (Fig. 3b and c), a commercial myelin probe (structure not disclosed or known). These results strongly suggest that **MyL-1** targeting substances in brain parenchymal is axonal myelinsheaths. It is worth noting that although FluoroMyelin™ Green (FMG) is commercially available, its chemical structure is unknown, as well as its use for super-resolution nanoscopy has never been described elsewhere.

### Photostability test of MyL-1 and its STED super resolution image of myelin ultrastructure

The network of myelin sheaths, and its physical parameters (e.g. thickness and diameter), both play a critical role in maintaining the normal function of the central nervous system electrical impulse. It remains of excellent significance for the neurologist to understand their ultrastructure under pathological or development conditions. **MyL-1,** as an optical probe capable of efficiently and accurately marking myelin sheaths with red emissive (emission = 700 nm). This offered the possibility to use **MyL-1** to construct myelin ultrastructure under STED dount laser (depletion = 775 nm).

The photostability of **MyL-1** was first assessed before performing the super-resolution imaging. The brain slices were stained with **MyL-1**, and then exposed to continued confocal and depletion laser irradiation over 100 scan time. The commercial probe FMG was used in parallel as a control. The fluorescent intensity over time (Additional file 1: Fig. S10) indicated that **MyL-1** fluorescence stays relatively stable after dense exposure, as over 95% and 80% of the signal was retained under confocal and STED depletion conditions, respectively. However, FMG displayed a sharp decrease in fluorescence, particularly upon STED irradiation, suggesting it is not practicable for such super-resolution-based evaluations.

**Fig. 4:**
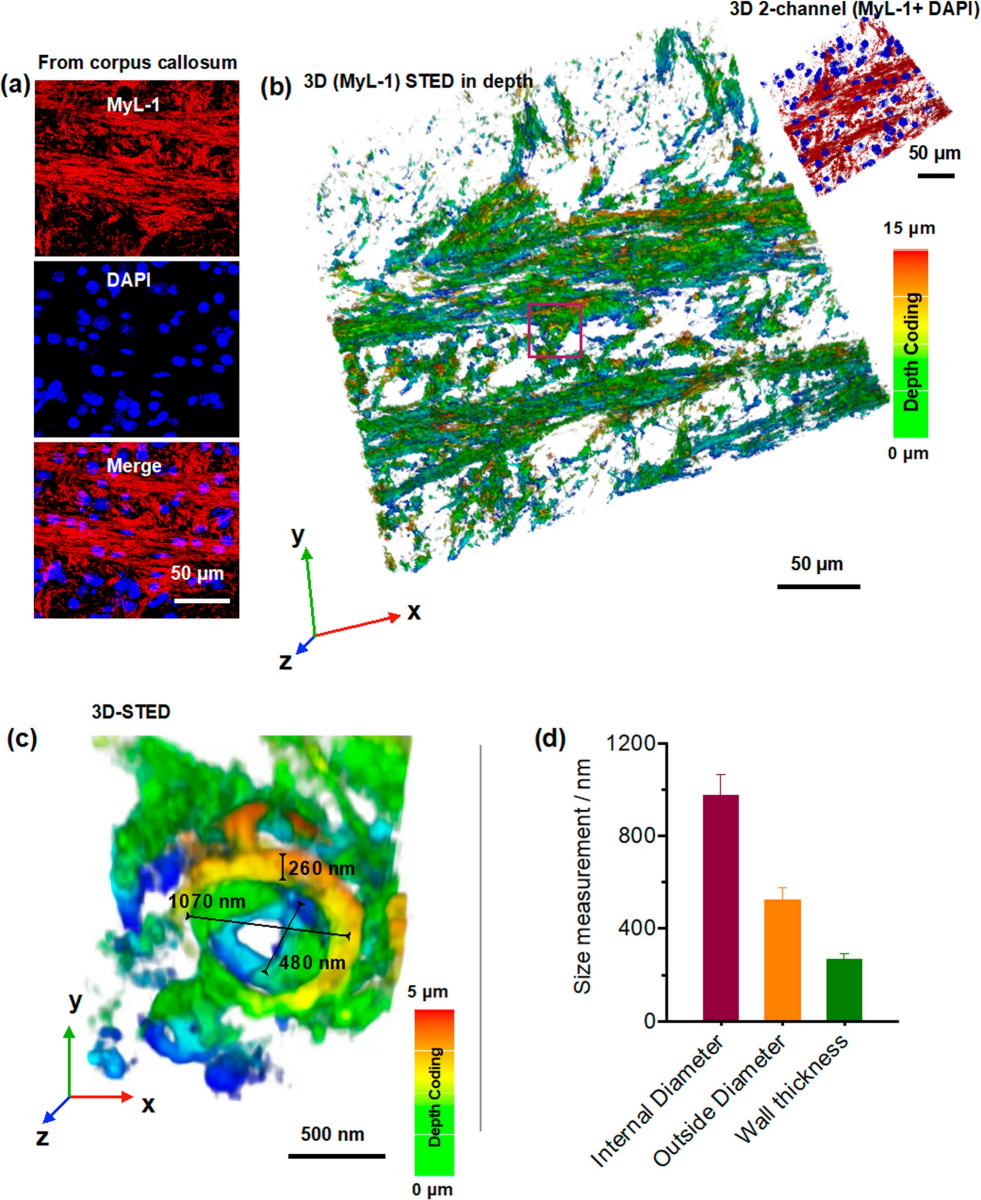
**a** Corpus callosum region from mouse brain section labeled by **MyL-1**, the nucleus were marked by DAPI. **b** Correspondent 3D-STED image of **MyL-1** labeled corpus callosum region displayed by depth coding (thickness ~ 15 μm, STED depletion = 775 nm). **c** 3D-STED image of selected region showed a myelinic tube structure by depth coding (thickness ~ 5 μm).at unprecedented resolution. **d** Quantification of **MyL-1** labeled myelinic tubular parameters including internal, outside diameter, and wall thickness (n = 10–20 images from three independent experiments)

Upon validating the **MyL-1** superior photon resistance under both confocal microscopy and STED nanoscopy, we 3D re-constructed the myelin ultrastructure. Neuron rich region corpus callosum was initially imaged under conventional confocal microscopy, and intense fibril patterns with parallel and vertical directions were detected (Fig. 4a). The correspondent 3D STED micrograph with depth-coding was subsequently captured (thickness = 15 μm, 89 STED slices), indicating an excellent spatial resolution (Fig. 4b). The magnified imaging at the selected region (from Fig. 4b) suggested that a single myelin sheaths tubular structure could be successfully visualized in 3D (Fig. 4c, left). Moreover, the super-resolution analyses enabled further resolution of more details with unprecedented quality, including myelinic internal/outside diameter and wall thickness. These parameters could also quantitatively be extracted from mature mouse brain (Fig. 4d).

### STED nanoscopic evaluation of myelin and MBP distribution at single axon level

**Fig. 5:**
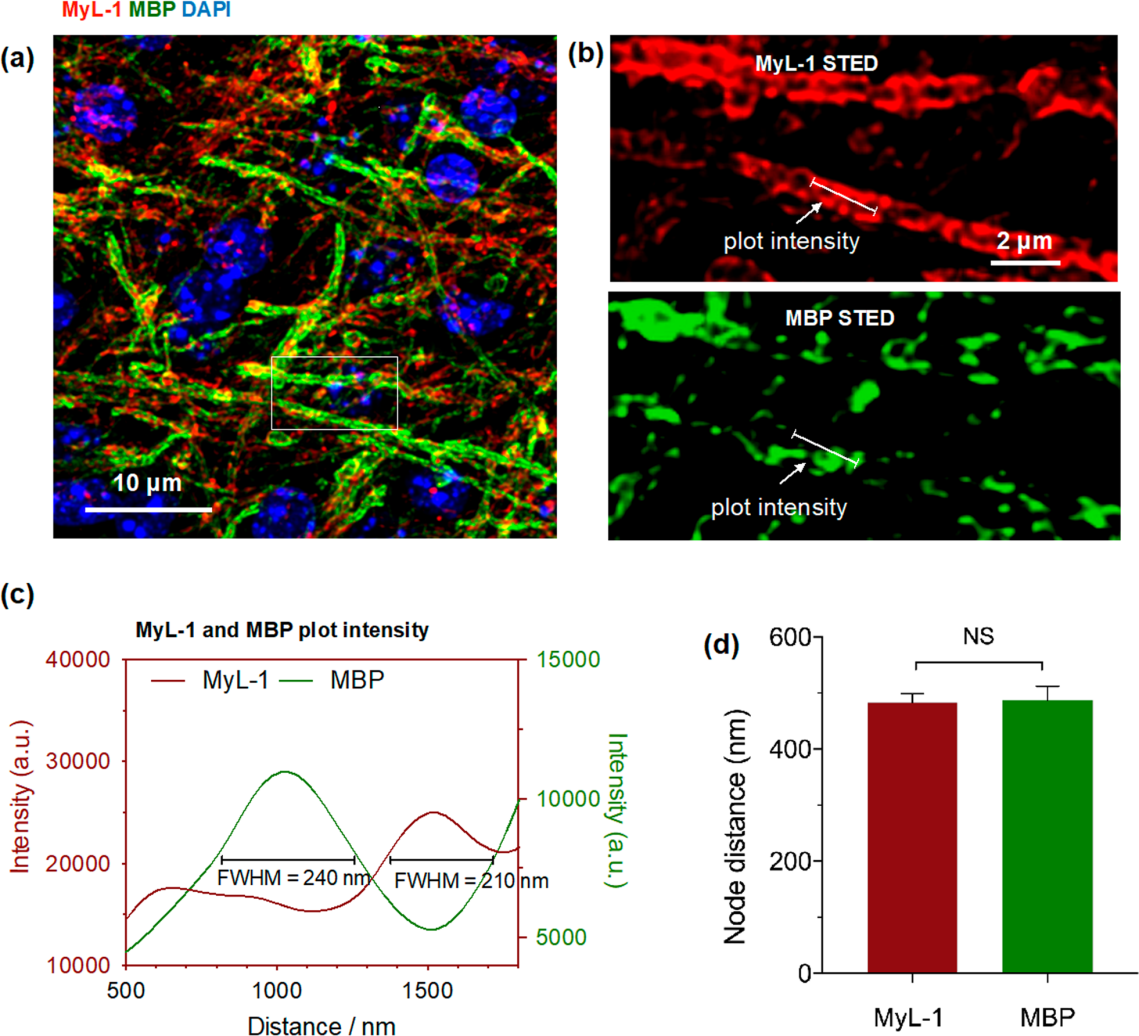
**a** Mouse brain section (corpus callosum region) incubated with **MyL-1** compound (20 μM) for 30 min, and immunofluorescently stained with MBP (ATTO 655 STED secondary antibody, STED depletion = 775 nm), the nucleus is marked by DAPI. **b** Magnified two-color STED imaging showed myelin and MBP distribution along a single axon. **c** **MyL-1** and MBP fluorescence intensity analysis along a selected region from **b** at a single axon. **d** Quantification of myelin (**MyL-1**) and MBP node distance along axons (n = 10–20 images from three independent experiments. Statistical analysis: student t-test, NS = No Significance)

After successfully using **MyL-1** to reconstruct myelin sheath in 3D ultrastructure, we further used this optical tool to explore the myelin association with MBP. MBP critically acts in the process of myelination of nerves during development, as well as in demyelinating disease. However, the dynamic information during myelinogensis, and especially the spatial relationship between myelin and MBP, have been rarely revealed. Herein, a mature mouse brain section was chosen and incubated with **MyL-1**, followed by immune-staining with MBP antibody (FITC labeled secondary antibody, excitation = 488 nm, Fig. 5a). The enlarged STED micrographs (Fig. 5a insert), together with the re-constructed 3D STED imaging, clearly revealed both myelin (**MyL-1**) and MBP at super-resolution. **MyL-1**-stained myelin signal indicated good compatibility with commercial fluorophores, even after multi-stages of fixation. Also, both myelin and MBP demonstrated a staggered distribution with similar node distance (~ 450 nm, Fig. 5c, d). This suggests that MBP can be used as an indirect method to confirm whether myelinic structure can have non-physiological patterns. The super-resolution nanoscopy results demonstrated that myelin and MBP have disparate locations below ~ 100 nm resolution, suggesting they might play different roles to maintain the axonal conductivity and possess diverse ‘time-axis’ during CNS development.

### Assessments of myelinogenesis during development and its correlations with MBP under STED nanoscopy

**Fig. 6:**
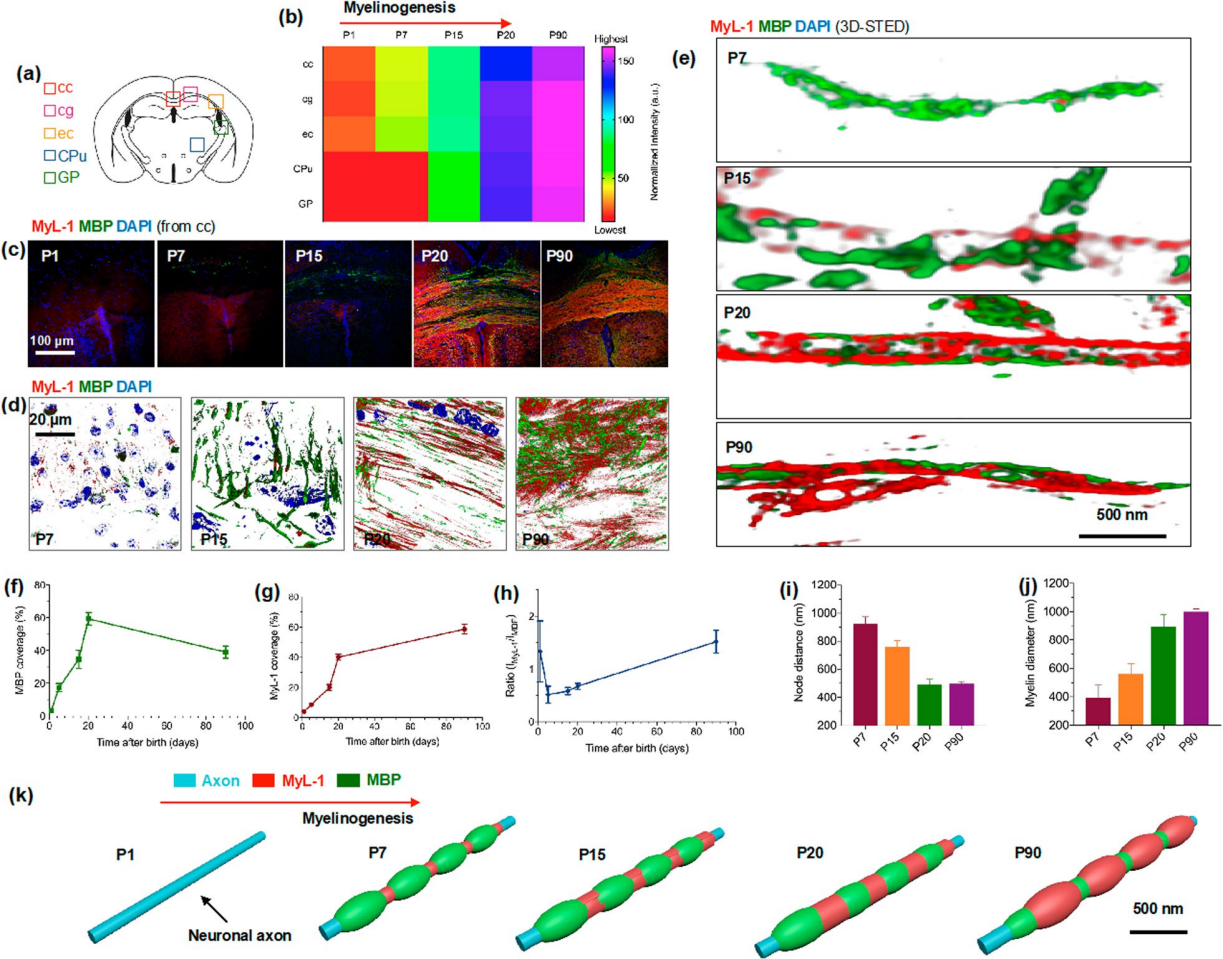
**a** Highlight of five different brain region cc (corpus callosum), cg (cingulum), ec (external capsule), CPu (corpus striatum), and GP (globus pallidus) under confocal and STED for myelinogenesis evaluation. **b** Brian section of P1, P7 P15, P20, and P90 mice stained with **MyL-1** and analysis of fluorescent intensity in cc, cg, ec, Cpu, and GP regions over times (n = 3). **c** Representative brain section of cc region at P1, P7, P15, P20, and P90, with MBP marked as green and nucleus marked as blue. **d** Magnified 3D confocal images of cc region at P7, P15, P20, and P90 showed myelin and MBP evolution (Note both myelin and MBP signal are hard to resolve at P1). I Correspondent two-color STED imaging showed myelin and MBP growing at single axon level over time. Fluorescence intensity quantification of MBP coverage (**f**) and myelin coverage (**g**) with their intensity ratio (I_MyL-1_/ I_MBP_) over times (**h**). Quantification of myelin node distance (**i**) and its diameter (**j**) at P7, P15, P20 and P90 (n = 10–15 images from three independent experiments). **k** Schematic representation of proposed myelinogenesis and its correlation with MBP during development

Motivated by the previous finding, we finally tried to investigate myelinogensis, including its relationship with MBP during development postnatally. The mouse brain sections were harvested at postnatal time of 1, 7, 15, 20, and 90 days (P1, P7, P15, P20, P90). Their neuronal axon rich regions (Fig. 6a) including corpus callosum (cc), cingulum (cg), external capsule (ec), corpus striatum (CPu), and globus pallidus (GP), were systematically evaluated under confocal microscopy and STED at the tissue- and single-axonal level, respectively. **MyL-1** fluorescent intensity was initially analyzed in these regions via multiple representative micrographs (Fig. 6c and Additional file 1: Figs. S11–S14). The overall myelinogenesis trend remains at a very low level (from P1 to P7) throughout the whole brain (Fig. 6b), while cc, cg, and ec demonstrated a more rapid development than CPu and GP. Moreover, myelin sheath formation increased significantly after P20 (Fig. 6b), and it is also suggested that myelination still keeps slow-growth tendency even at P90.

The brain sections were also immuno-labelled (with MBP antibody) at different postnatal time points, in different regions, to study the correlations between myelin and its associate protein during myelinogenesis. The corresponding high-resolution confocal 3D micrographs and STED 3D images were subsequently captured and quantitatively investigated. Figure 6d displayed the evolution of MBP, as well as **MyL-1** marked myelin sheath from P7 to P90 (from cc region). Representative two-colors (MBP and **MyL-1**) super-resolution images are also displayed (Fig. 6e). Interestingly, MBP has a sharp increase at first 20 days postnatally, then it gradually decreases to an approximate half volume at P90 (Fig. 6f). During this process, myelin sheath volume demonstrated a sustainable growth with the appearance of repeated node structure (Fig. 6g). Therefore, the volume ratio of myelin (**MyL-1**)-to-MBP indicated a decrease first (P1–P20), followed by an increase (P20–P90). It is also noticeable that the node distance (the distance between two distinct signals on axons) could reach a mature level at P20 (~ 500 nm), while the myelin diameter showed a consistent expansion even at P90.

The above results identified two critical interactions between MBP and myelin sheath during myelinogenesis (Fig. 6k). Firstly, MBP appearance is approximately two weeks earlier than myelin on axon postnatally, and it might play a role of ‘guiding’ the growth and packing of the myelin sheath. Secondly, upon directing myelin building on the axon, the constituents of MBP would be dropped to a lower extent compared to its peak level, to ‘give up’ more space for myelin sheath development. Nonetheless, to the best of our knowledge, this is the first time that the dynamic relationships between MBP and myelin are demonstrated during CNS evolution under super-resolution nanoscopy.

## Materials and methods

### Materials and apparatus

All chemical ingredients were obtained commercially. Solvents were purified with conventional methods before use. The ^1^H (600 MHz) NMR spectra were collected on a Bruker Avance 400 spectrometer at 25 °C (TMS as the internal standard in NMR). Coupling constants J were given in Hertz. Mass spectra were performed on a Micromass GCT-MS (ESI source) and MALDI-TOF–MS. Absorption spectra were recorded on a UV-3100 spectrophotometer. Emission spectra were obtained on an F-2500 fluorescence spectrophotometer.

### X-ray crystallography and structure solution

X-ray diffraction data of single crystals were collected by Siemens Smart 1000 CCD diffractometer, and the determination of unit cell parameters and data collections were performed with MoK_α_ radiation (λ = 0.71073 Å). Unit cell dimensions were collected with least-squares refinements and all structures were solved by direct methods using SHELXS-97. The other non-hydrogen atoms were located in successive difference Fourier syntheses. The final refinement was performed by full-matrix least-squares methods with anisotropic thermal parameters for non-hydrogen atoms on F^2^. The hydrogen atoms were added theoretically and riding on the concerned atoms.

### Computational theoretical calculation

To better understand the charge transfer state, time-dependent density functional theory (TD-DFT) calculations on all the compounds were carried out in THF. Optimizations were carried out with B3LYP functional without any symmetry restraint, and the TD-DFT calculations were performed on the optimized structure with B3LYP functional. All calculations, including optimizations and TD-DFT, were performed with the G09 software. Geometry optimization of the singlet ground state and the TDDFT calculation of the lowest 25 singlet–singlet excitation energies were calculated with a basis set composed of 6–31 G* for C H N O P F atoms. An analytical frequency confirms evidence that the calculated species represents a true minimum without imaginary frequencies on the respective potential energy surface. The lowest 25 spins allowed singlet–singlet transitions, up to energy of about 5 eV were taken into account in the calculation of the absorption spectra.

### Animals and brain sections

All procedures involving animals were approved by and conformed to the guidelines of the West China Hospital Animal Care Committee. We have made great efforts to reduce the number of animals used in these studies and also made efforts to reduce animals suffering from pain and discomfort. The brain tissue was isolated from P1, P7, P15, P20, P90 Specific pathogen Free (SPF) Kumming (KM) mice after euthanasia. Tissues were put into 4% paraformaldehyde for 3–5 days and then dehydrated in 30% sucrose solution. The fixed mouse brain slices (20 μm) were obtained by Leica CM3050S freezing microtome. The slices were stained with complex **MyL-1** (20 μM) for 30 min at room temperature. The imaging was performed after the slices were washed by PBS 3 times. To avoid misclassification due to individual differences in animals, at least three independent replicates were set up for each animal experiment.

### Immunofluorescence

Prefixed tissue was applied 0.5% Triton X-100 for 5 min and washed with PBS 3 times for 5 min every time. After incubating with 100 mM glycine for 15 min at room temperature, PBS washed the cell again for 5 min every time. Posteriorly, the cell was covered with 1% bovine serum albumin (BSA) for 1 h in order to close the non-specific binding sites for primary antibodies (Abcam, ab216590 for MBP; Thermo fisher MA5-47125 for MAP2; Thermo fisher, PA5-78499 for NeuN; Abcam, and ab7260 for GFAP) and incubated by using primary antibodies in the 4-degree refrigerator for more than 12 h. After washing with PBS 3 times for 10 min every time, incubated with fluorescent secondary antibodies (Thermo fisher, A-11008 and Abcam, ab150078) for 1 h without exposure to light. The imaging was carried out after the cells were washed by PBS 3 times.

### Confocal imaging

Confocal microscopy imaging was acquired with a Leica TCS SP8 confocal microscopy and 20X objective lens, 63X/100X oil-immersion objective lens. The incubated slices were excited at 600 nm for **MyL-1** for one photon imaging, 405 nm for DAPI, 495 nm for Lectin, 488 nm for fluorescent secondary antibodies with a semiconductor laser, and the emission signals were collected at 700 ± 20 nm for **MyL-1**, 430 ± 20 nm for DAPI, 515 ± 20 nm for Lectin, 520 ± 20 nm for fluorescent secondary antibodies, respectively.

### STED imaging

STED nanoscopy experiments were performed under Leica DMi8 confocal microscopy equipped with Leica TCS SP8 STED-ONE unit and the compound was excited under STED laser, the emission signals were collected using HyD reflected light detectors (RLDs). Specimen living cells were prepared using a similar method as normal confocal microscopy described previously, and donut laser was used in 775 nm STED laser (50% power), with 2048*2048 pixels and *100 scanning speed. The STED micrographs were further processed ‘deconvolution wizard’ function using Huygens Professional software (version: 16.05) under an authorized license. The area radiuses were estimated less than 0.02 micros with the exclusion of 100 absolute background values.

### Image processing and analysis

Micrographs were processed and analyzed by Huygens software and ImageJ 1.48 v (32-bit). Quantification of the fluorescence intensity was achieved via Analyze >  > Tools >  > ROI manager in ImageJ from three parallel experiments. Quantification of single myelin intensity profile was achieved via Analyze >  > Plot Profile by selecting one myelin in ImageJ. Quantification of colocolization coefficiency was achieved via an external plugin via Plugins >  > Colocolization Finder. For more details, please refer to online sources: https://imagej.nih.gov/ij/

## Conclusion

Serials of curcumin BODIPY like compounds **MyL-1**, **MyL-2,** and **MyL-3** were rationally designed for ex vivo myelinic targeting. It was found one of the compounds **MyL-1** possesses near infrared emission with a larger stock shift, and capable of targeting sphingomyelin with 16 folds fluorescence enhancement. We showed that **MyL-1** can successfully label myelinic substances in brain models with high specificity, and can undergo harsh STED nanoscopic challenges, due to its excellent photostability. It is further successfully demonstrated that **MyL-1** can directly highlight myelin ultrastructure under STED nanoscopy during myelinogenesis, and provide a valuable clue about the correlation between myelin and MBP along with vertebral development. This work offered a straightforward method to visualize myelinic ultra details and anticipate to assist in better answering correlated cognitive, motor skills, and demyelinating questions.

### Supplementary Information

**Additional file 1.** Additional synthesis and characterization of **MyL-1**, **MyL-2**, and **MyL-3 (Fig. S1-Fig. S6)**. Molecular orbital energy of **MyL** **(Fig. S7)**. Sphingomyelins bilayer interaction with **MyL** **(Fig. S8)**. Cytotoxicity data results of **MyL-1**, **MyL-2 **and **MyL-3** **(Fig. S9)**. Photostability evaluation of **MyL-1** **(Fig. S10)**. Confocal, and its magnified 3D-STED images of tissue sections treated with **MyL-1 (Fig. S11-Fig. S14)** and Corresponding photophysical data **(Table S1)**.

## Data Availability

All data generated or analyzed during this study are included in this published article and the supplementary information.
