# Supplementary material for: Nanoscale myelinogenesis image in developing brain via super-resolution nanoscopy by near-infrared emissive curcumin-BODIPY derivatives
Source: J Nanobiotechnology. 2024 Mar 11;22:106. doi: 10.1186/s12951-024-02377-9 (PMC10929150; doi:10.1186/s12951-024-02377-9)
Supplement: Supplementary file 1 — Additional file 1. Additional synthesis and characterization of MyL-1, MyL-2, and MyL-3 (Fig. S1-Fig. S6). Molecular orbital energy of MyL (Fig. S7). Sphingomyelins bilayer interaction with MyL (Fig. S8). Cytotoxicity data results of MyL-1, MyL-2 and MyL-3 (Fig. S9). Photostability evaluation of MyL-1 (Fig. S10). Confocal, and its magnified 3D-STED images of tissue sections treated with MyL-1 (Fig. S11-Fig. S14) and Corresponding photophysical data (Table S1). [file 12951_2024_2377_MOESM1_ESM.docx]

### Additional file

**Nanoscale Myelinogenesis Image in Developing Brain via Super-resolution Nanoscopy by Near-Infrared Emissive Curcumin-BODIPY Derivatives**

Junyang Chen, ^a, b, d, g, h 1^ Yifan Yu, ^b, 1^ Siyou Wang, ^d^ Yu Shen, ^d^ Yupeng Tian, ^d^ Loris Rizzello, ^e, f^ Kui Luo, ^b^ Xiaohe Tian, ^a, b, h^ * Tinghua Wang, ^a, c^ * Liulin Xiong, ^a^*

^a^ *Department of Anesthesiology, Affiliated Hospital of Zunyi Medical University No.149, Dalian Road, Huichuan District, Zunyi 563000, Guizhou, China*

^b^ *Functional and Molecular Imaging Key Laboratory of Sichuan Province, Huaxi MR Research Centre (HMRRC), Department of Radiology and National Clinical Research Center for Geriatrics, West China Hospital of Sichuan University, Chengdu, 610000, China*

^c^ *Institute of Neurological Disease, Translational Neuroscience Center, West China Hospital, Sichuan University, Chengdu 610041, China*

^d^ *Department of Chemistry, Key Laboratory of Functional Inorganic Material Chemistry of Anhui Province, Anhui University, Hefei, 230601, China*

^e^ *Department of Pharmaceutical Sciences, University of Milan, Via G. Balzaretti 9 - 20133 Milan, Italy*

*^f^ The National Institute of Molecular Genetics (INGM), Via Francesco Sforza 35 - 20122 Milan, Italy*

^g^ *Department of Chemistry, University College London, WC1H 0AJ, London, United Kingdom*

*h Laboratory of Aging Research and Cancer Drug Target, State Key Laboratory of Biotherapy and Cancer Center, West China Hospital, Sichuan University, Chengdu 610041, China.*

________________________________________________________________________________

*Corresponding authors: Liulin Xiong [(liulin.xiong@mymail.unisa.edu.au),](mailto:(liulin.xiong@mymail.unisa.edu.au),) Tinghua Wang [(wangtinghua@vip.163.com)](mailto:(wangtinghua@vip.163.com)) and Xiaohe Tian [(xiaohe.t@wchscu.cn)](mailto:(xiaohe.t@wchscu.cn),)

^1^ These authors contributed equally to this work as co-first author.

**Synthesis and characterization**

The synthetic routes of **MyL-1**, **Myl-2** and **MyL-3** are shown in Scheme S1-S3. They were prepared base on previous work with minor modifications [1] and according to the similar procedures with the references [2]. Compounds **a** and **c** were synthesis based on previous work with minor modifications [3]. Compounds **b** and **d** were prepared according to the similar procedures with the references [4].

**Synthesis of MyL-1**


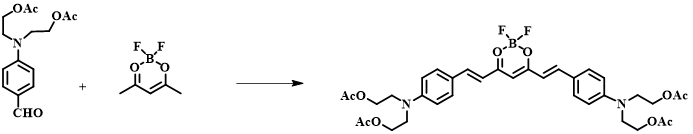


**a b MyL-1**

**Scheme S1****.** Synthetic procedures for probe **MyL-1**.

**MyL-1. a** 2.90 g (10 mmol) was dissolved in refined toluene and **b** 0.75 g (5 mmol) was added in batches. Then tributylborate 2.50 mL (10.05mmol) and n-butylamine 0.30 ml were added. The reaction was reacted at 60 °C for 10 h. After cooling to room temperature, red solid powder was precipitated out and filtered. The powder was washed by hot toluene, and then filtered while hot. After vacuum drying, the dark red powder was obtained. ^1^H-NMR (400 MHz, d_6_-[Acetone](C://Users/Administrator.WIN7U-20150625I/AppData/Local/Youdao/Dict/Application/6.3.69.8341/resultui/frame/javascript:void(0);), ppm) δ 8.14 (dd, *J* = 16.3, 11.6 Hz, 3H), 7.90 – 7.71 (m, 3H), 7.63 (t, *J* = 7.7 Hz, 2H), 7.23 (s, 1H), 6.95 (dd, *J* = 12.2, 5.3 Hz, 3H), 4.22 (t, *J* = 5.7 Hz, 4H), 3.76 (t, *J* = 5.7 Hz, 4H), 1.99 (s, 6H). ESI m/z, calcd: 485, found: 508 ([M]+Na^+^).

**Synthesis of MyL-2**


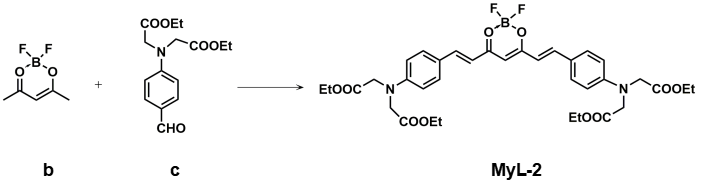


**Scheme S2.** Synthetic procedures for probe **MyL-2**.

**MyL-2** was prepared according to the similar procedures with **MyL-1** except that **a** was replaced by c^1^H NMR (600 MHz, CD_3_CN) δ 9.18 (d, J = 5.7 Hz, 2H), 8.24 – 8.21 (m, 2H), 8.19 – 8.15 (m,2H), 7.80 (s, 1H), 7.79 (s, 1H), 7.62 – 7.59 (m, 2H), 7.55 (dd, J = 8.1, 1.6 Hz, 2H), 6.67 (d, J = 1.6 Hz, 2H), 3.59 – 3.54 (m, 2H), 1.99 (s, 3H), 1.19 – 1.09 (m, 3H). ESI-MS: cal: 670.72, found: 670.58 [M−PF6^-^].

**Synthesis of MyL-3**


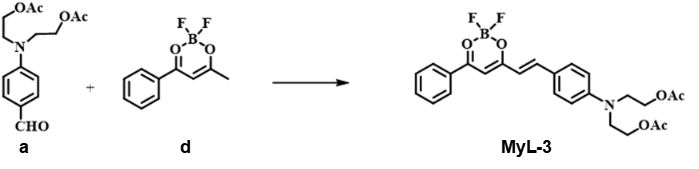


**Scheme S3.** Synthetic procedures for probe **MyL-3**.

**MyL-3** was prepared according to the similar procedures with **MyL-1** except that **b** was replaced by **d**.^1^H-NMR (400 MHz, d_6_-[Acetone](file:///C:/Users/Administrator.WIN7U-20150625I/AppData/Local/Youdao/Dict/Application/6.3.69.8341/resultui/frame/javascript:void(0);), ppm) δ 8.14 (dd, *J* = 16.3, 11.6 Hz, 3H), 7.90 – 7.71 (m, 3H), 7.63 (t, *J* = 7.7 Hz, 2H), 7.23 (s, 1H), 6.95 (dd, *J* = 12.2, 5.3 Hz, 3H), 4.22 (t, *J* = 5.7 Hz, 4H), 3.76 (t, *J* = 5.7 Hz, 4H), 1.99 (s, 6H). ESI m/z, calcd: 485, found: 508 ([M]+Na^+^).


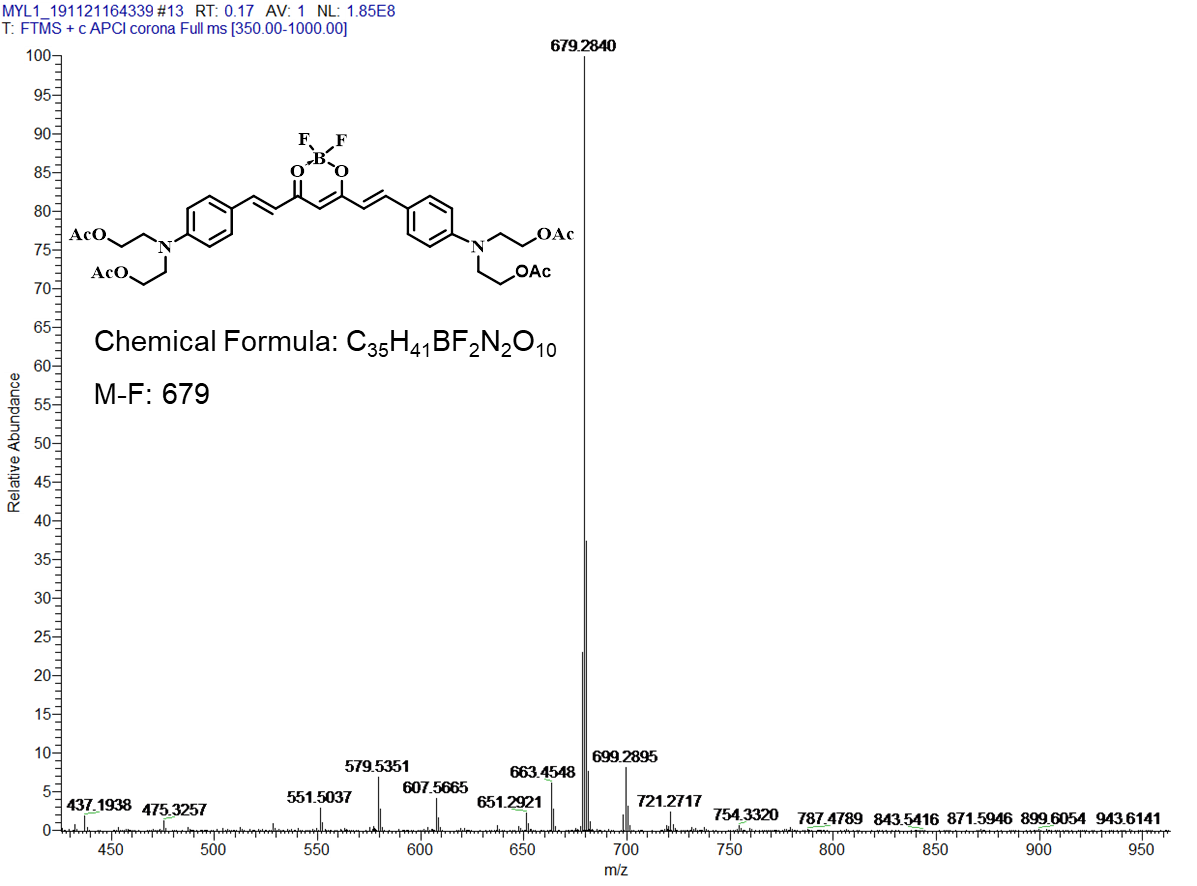
**Characterization of MyL-1**


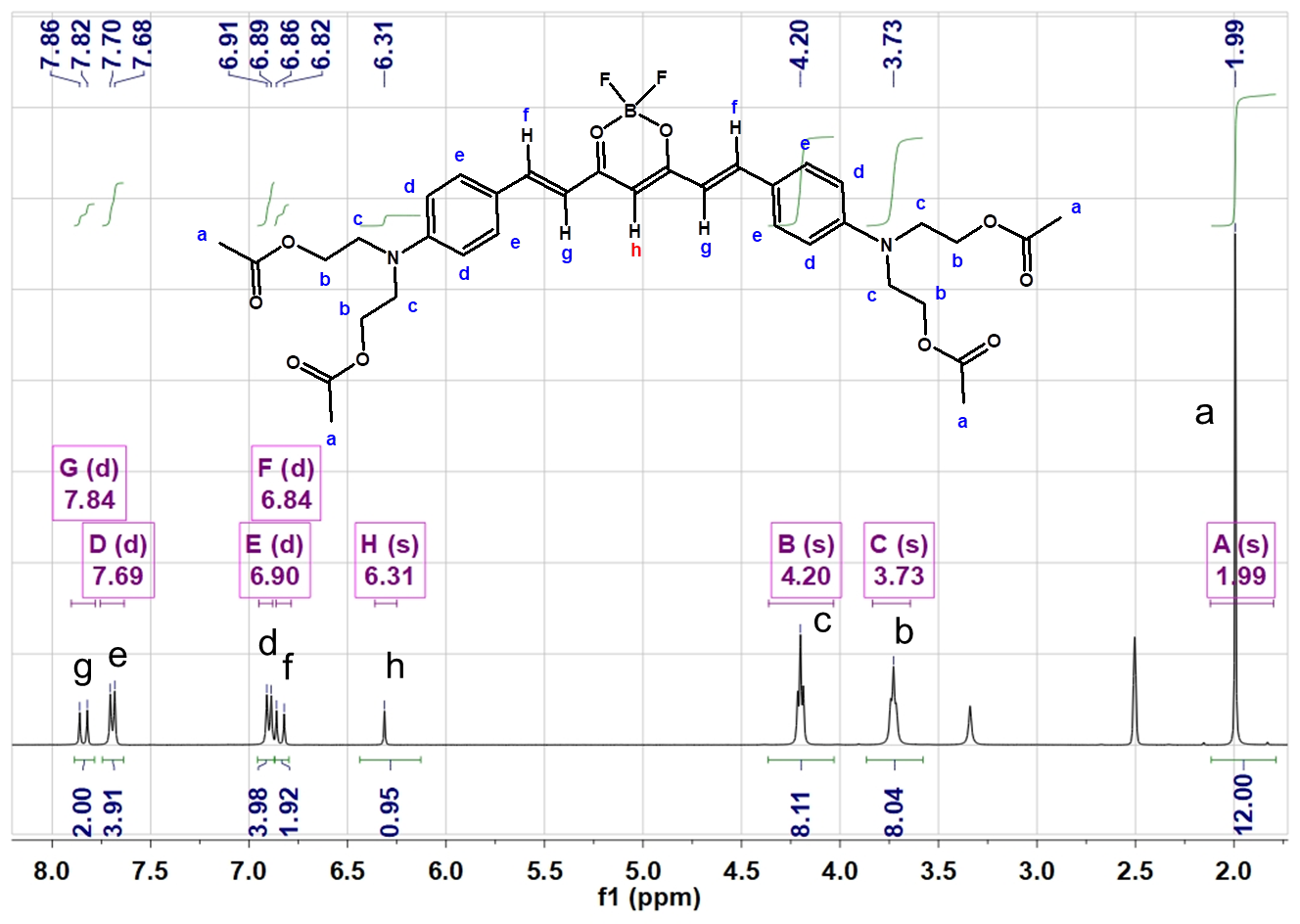
**Fig. S1** Mass spectra of **MyL-1**.

**Fig. S2** ^1^H NMR spectrum (600 MHz, CD_3_CN) of **MyL-1**.


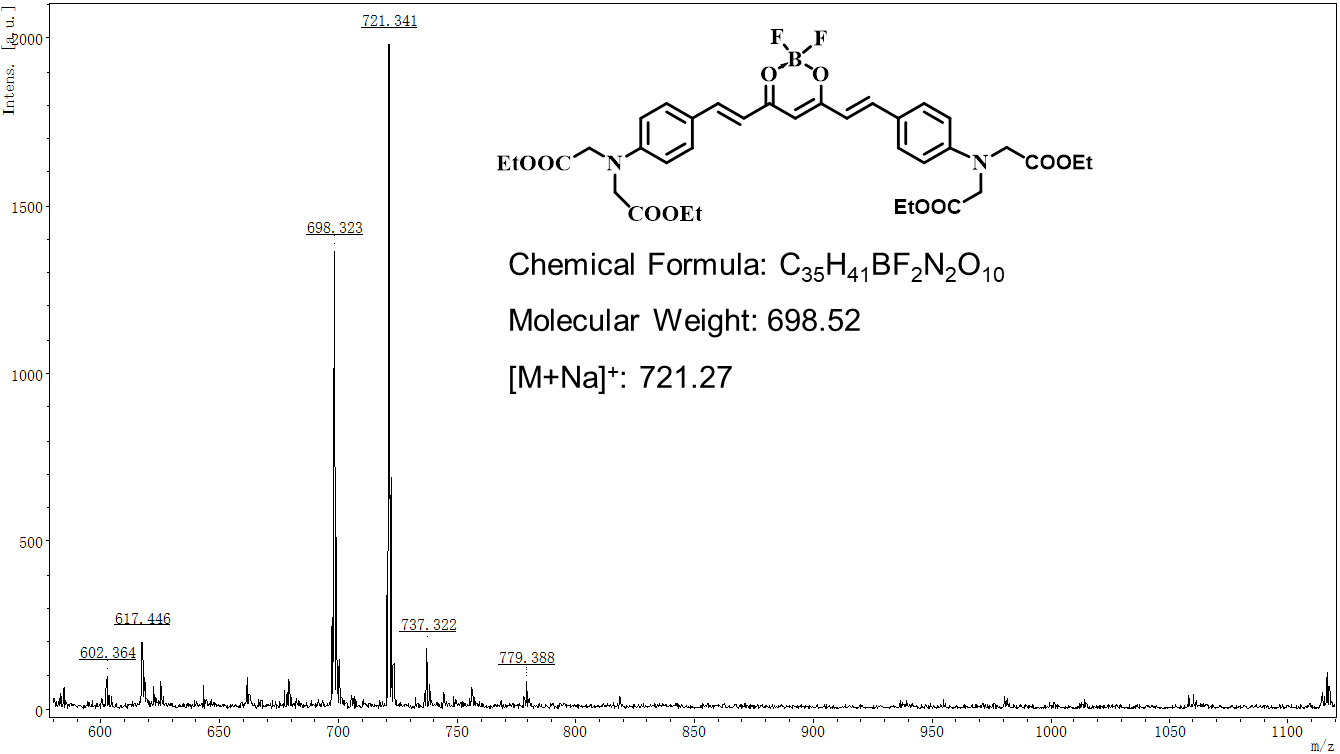
**Characterization of MyL-2**

**Fig. S3** Mass spectra of **MyL-2**.


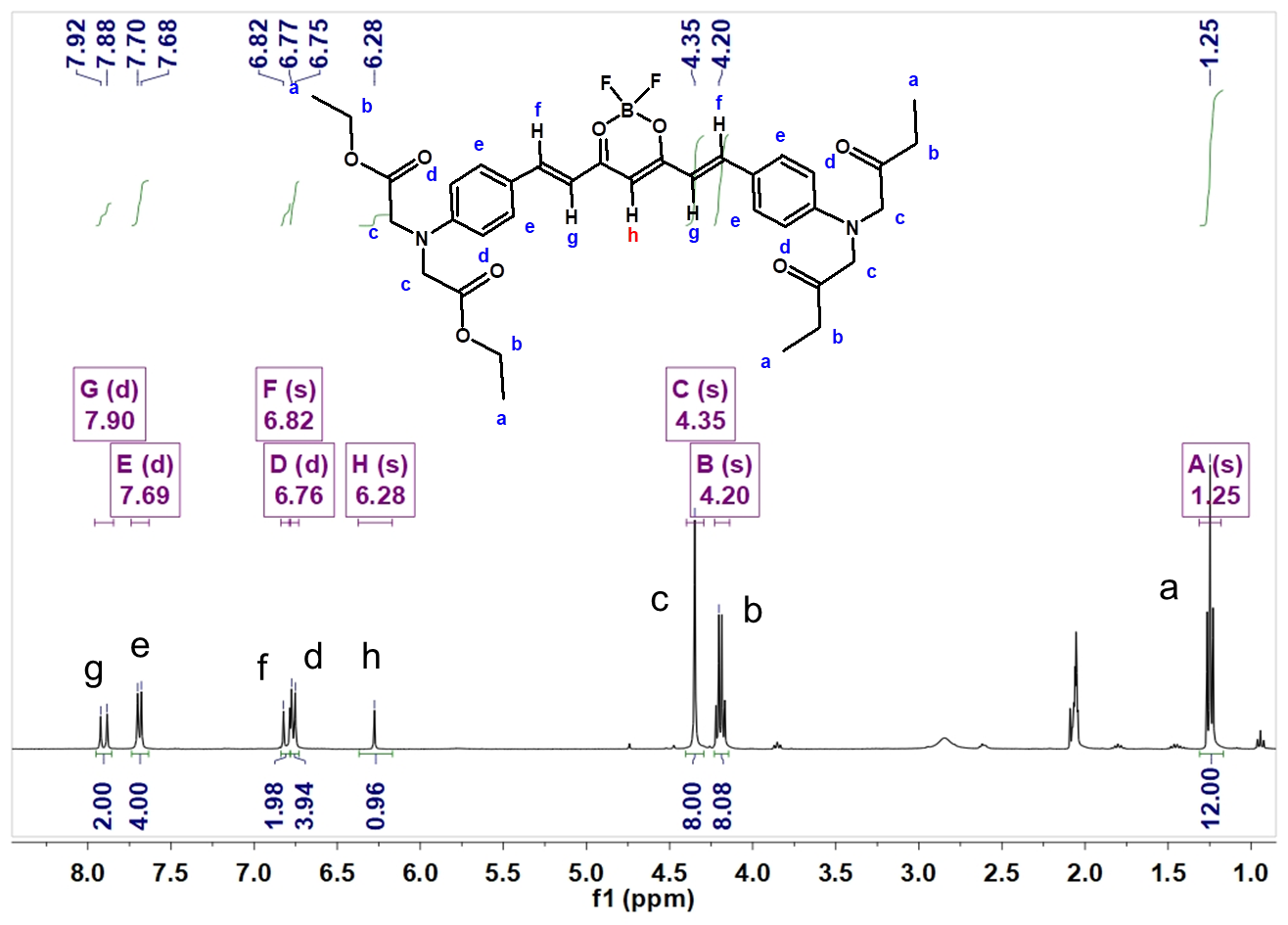


**Fig. S4** ^1^H NMR spectrum of **MyL-2**.


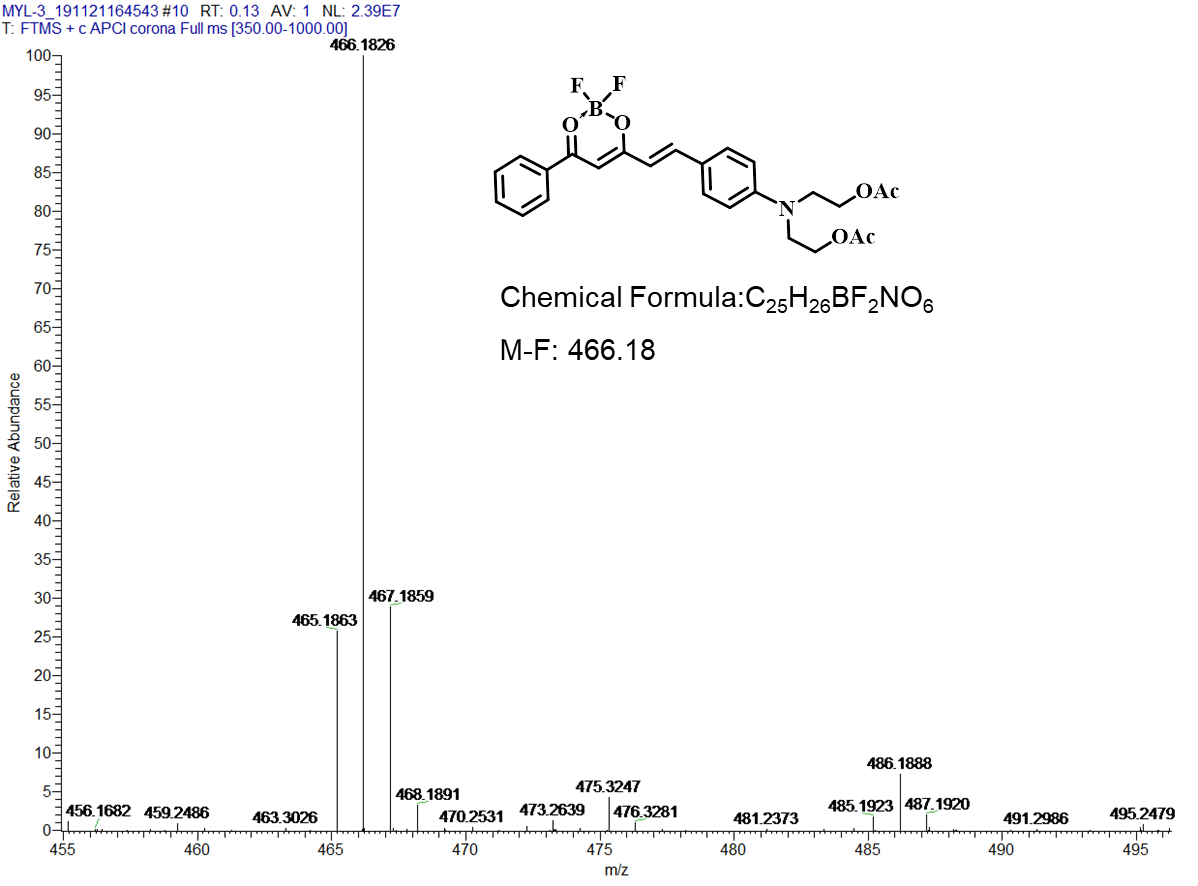
**Characterization of MyL-3**


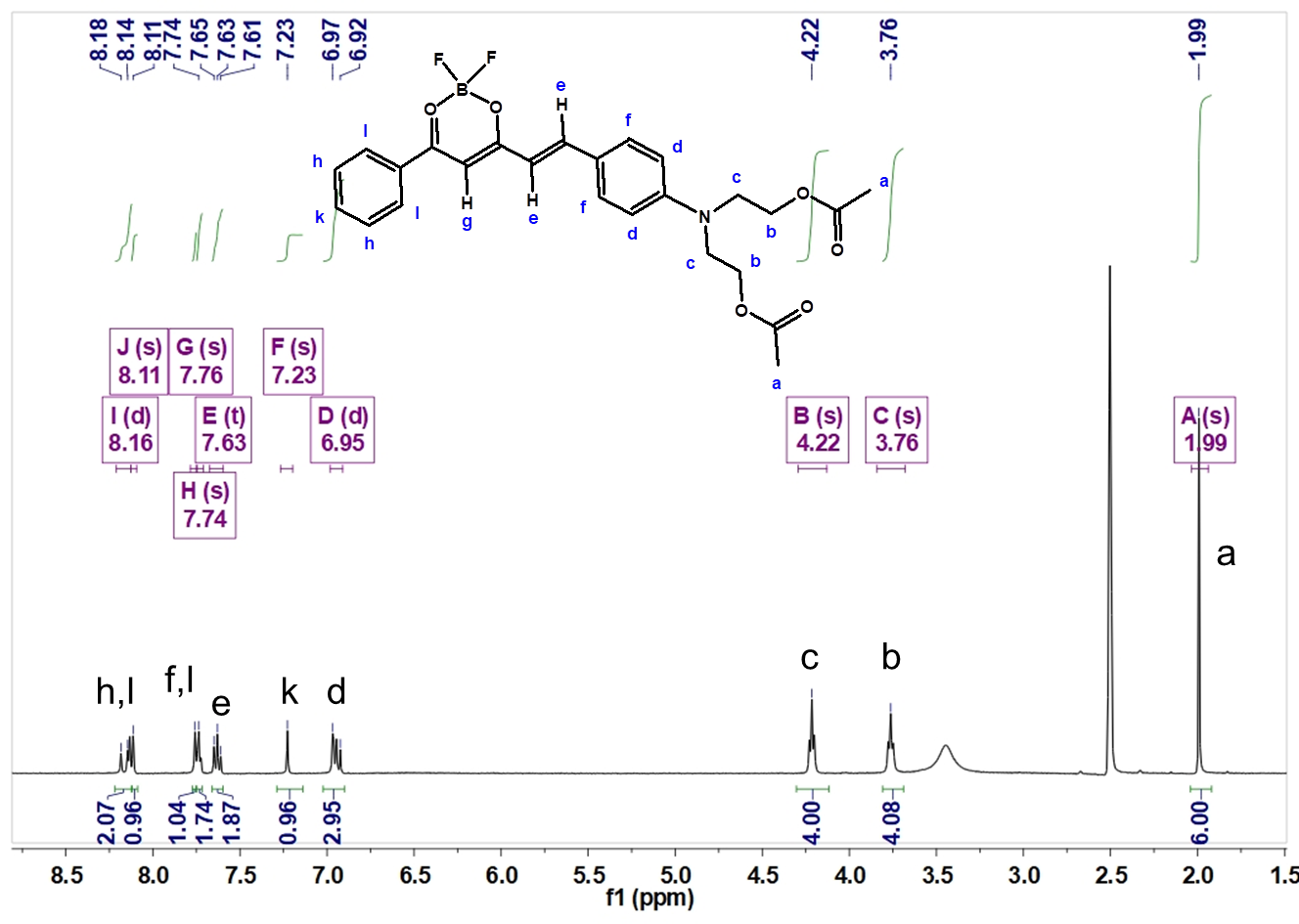
**Fig. S5** Mass spectra of **MyL-3**.

**Fig. S6** ^1^H NMR spectrum of **MyL-3**.

**Molecular orbital energy of MyL**


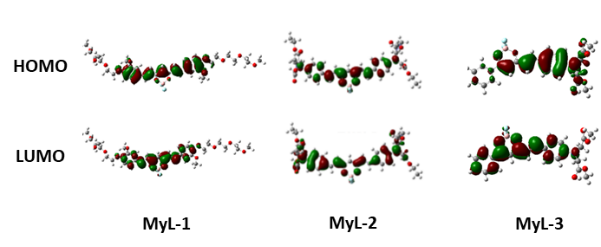


**Fig. S7** Molecular orbital energy diagrams for **MyL-1**, **MyL-2** and **MyL-3**.

**Sphingomyelins bilayer interaction with MyL**


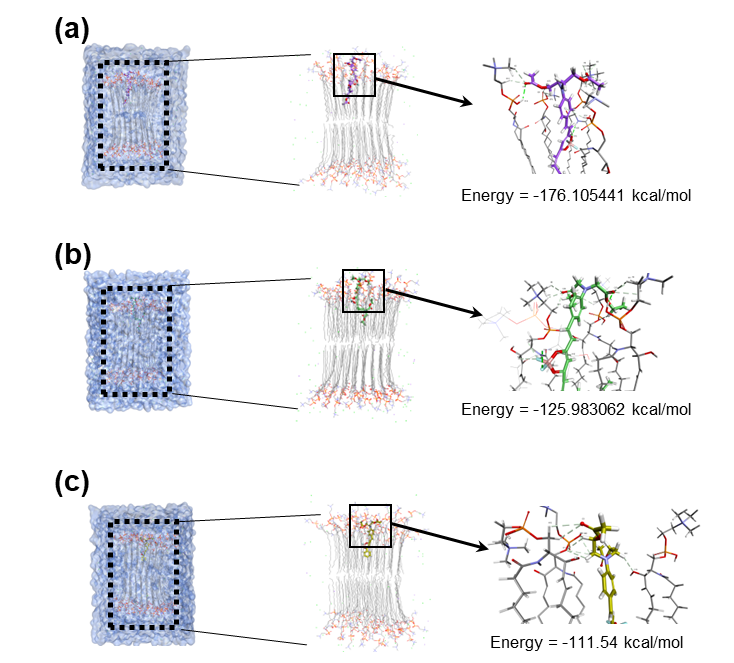
**Fig. S8** Sphingomyelins bilayer setup and simulation of **MyL** interaction. Interaction between molecule MyL-1 (a), MyL-2 (b), MyL-3 (c) and sphingomyelin.

**Cytotoxicity results**


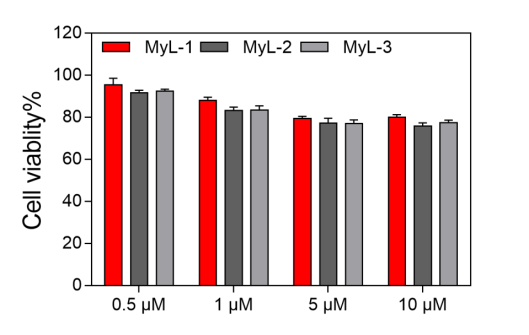


**Fig. S9** Cytotoxicity data results of **MyL-1**, **MyL-2**and **MyL-3** obtained from the MTT assay.

**Photostability evaluation of MyL-1**


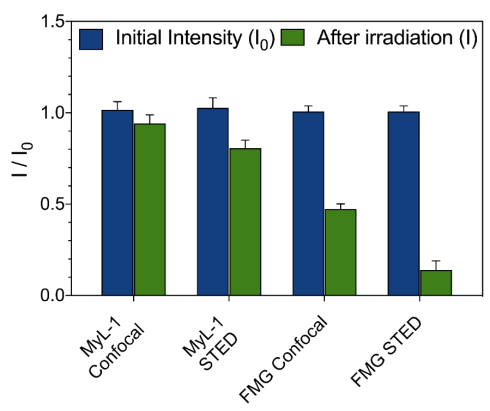


**Fig. S10** Photostability evaluation of **MyL-1** (excitation= 640 nm, emission = 700 nm, depletion laser = 660 nm) and commercialized FluoroMyelin^TM^ Green (excitation= 488 nm, emission = 520 - 550 nm, depletion laser = 592 nm) confocal and its magnified 3D-STED images of mouse brain tissue section at cingulum region, treated with probe **MyL-1** (20 μM). I: intensity, I0: solvent intensity.

**Confocal and its magnified 3D-STED images of tissue sections treated with MyL-1**


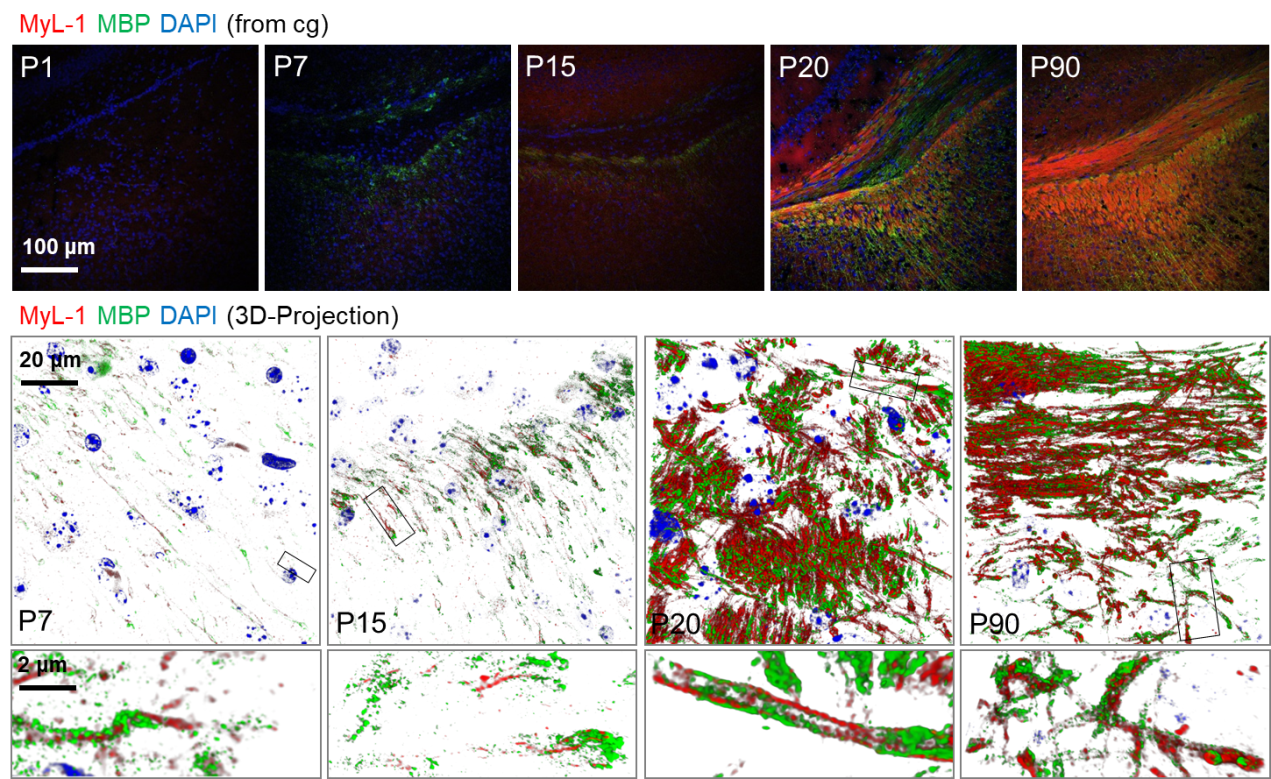
**Cingulum region**

**Fig. S11** Confocal and its magnified 3D-STED images of mouse brain tissue section at cingulum region at postnatal time of 1, 7, 15, 20, and 90 days, treated with probe **MyL-1** (20 μM), myelin based protein (MBP) marked as green and nucleus marked as blue.


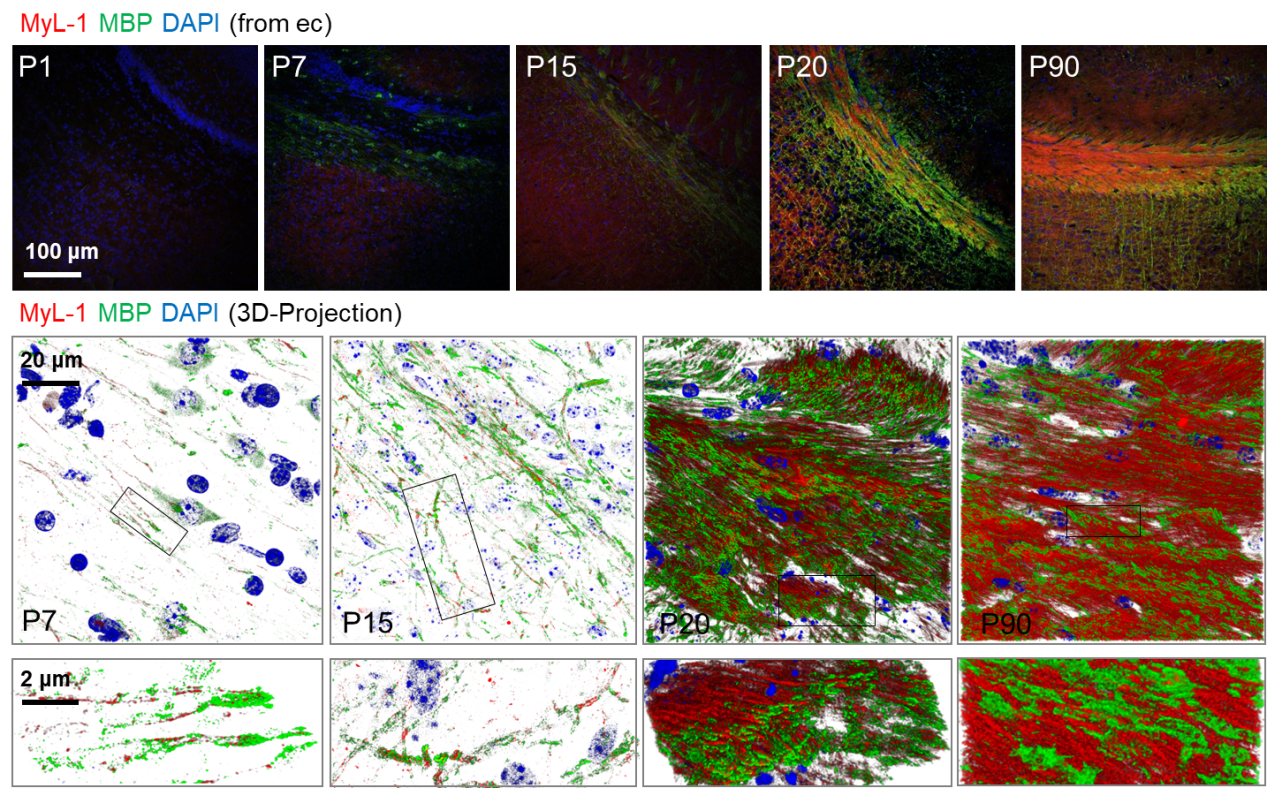
**External capsule region**

**Fig. S12** Confocal and its magnified 3D-STED images of mouse brain tissue section at external capsule region at postnatal time of 1, 7, 15, 20, and 90 days, treated with probe **MyL-1** (20 μM), myelin basic protein (MBP) marked as green and nucleus marked as blue.


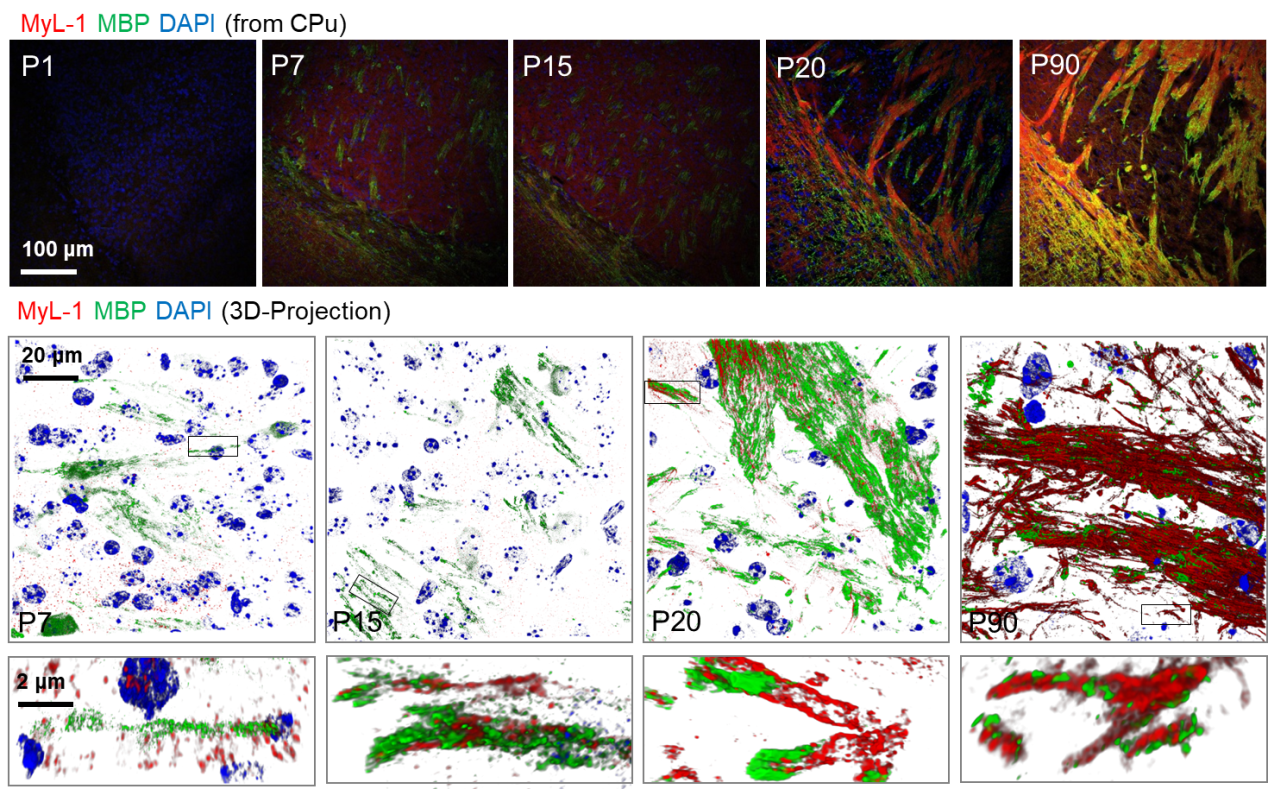
**Corpus striatum region**

**Fig. S13** Confocal and its magnified 3D-STED images of mouse brain tissue section at corpus striatum region at postnatal time of 1, 7, 15, 20, and 90 days, treated with probe **MyL-1** (20 μM), myelin based protein (MBP) marked as green and nucleus marked as blue.


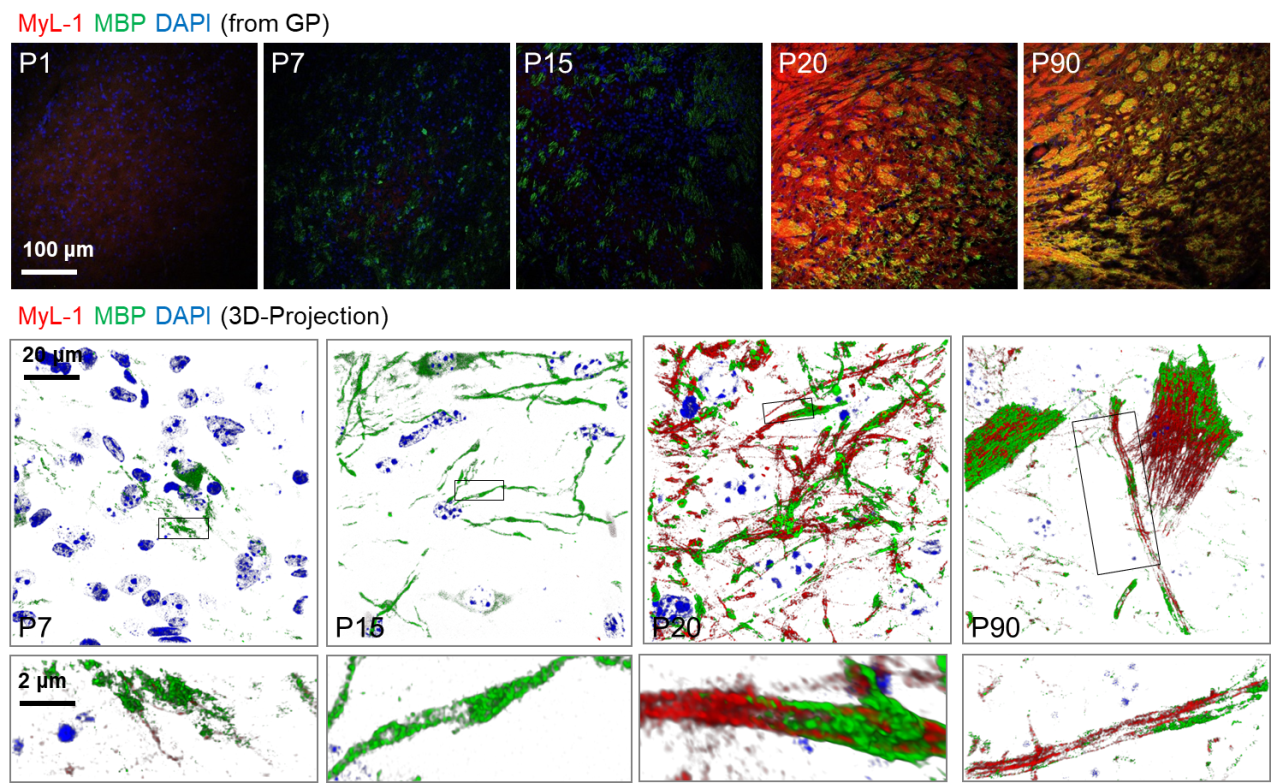
**Globus pallidus region**

**Fig. S14** Confocal and its magnified 3D-STED images of mouse brain tissue section at globus pallidus region at postnatal time of 1, 7, 15, 20, and 90 days, treated with probe **MyL-1** (20 μM), myelin based protein (MBP) marked as green and nucleus marked as blue.

**Corresponding photophysical data**

**Table S1.** The corresponding photophysical data of **MyL-1**, **MyL-2** and **MyL-3** in DMSO.

| **Compounds** | **Solvents** | ***λ*_max_^a^** | ***λ*_ma_^b^** | ***Φ*_f_^e^** | **τ/ns^f^** |
| --- | --- | --- | --- | --- | --- |
| **MyL-3** | DMSO | 542 | 646 | 13.21 | ﹤1 |
| **MyL-1** | DMSO | 607 | 695 | 30.12 | ﹤1 |
| **MyL-2** | DMSO | 584 | 668 | 68.36 | ﹤1 |

a Peak position of the largest absorption band in nm (10 μM). b Peak position of SPEF, exited at the absorption maximum. c Maximum molar absorbance in 10^4^ L/mol·cm. d Stokes shift in nm. e Quantum yields were the absolute quantum yields. f The fitted fluorescence lifetime.

**REFERENCES**

1. Zhao, M.; Zhu, Y.; Su, J.; Geng, Q.; Tian, X.; Zhang, J.; Zhou, H.; Zhang, S.; Wu, J.; Tian, Y. A water-soluble two-photon fluorescence chemosensor for ratiometric imaging of mitochondrial viscosity in living cells. J Mater Chem B. 2016, 4, 5907-5912.
2. Nakata, E.; Koizumi, M.; Yamashita, Y.; Onaka, K.; Sakurai, Y.; Kondo, N.; Ono, K.; Uto, Y.; Hori, H. Design, synthesis and destructive dynamic effects of BODIPY-containing and curcuminoid boron tracedrugs for neutron dynamic therapy. Anticancer Res. 2011, 31, 2477-2481.
3. Zhao, M.; Zhu, Y.; Su, J.; Geng, Q.; Tian, X.; Zhang, J.; Zhou, H.; Zhang, S.; Wu, J.; Tian, Y. A water-soluble two-photon fluorescence chemosensor for ratiometric imaging of mitochondrial viscosity in living cells. J Mater Chem B. 2016, 4, 5907-5912.
4. Nakata, E.; Koizumi, M.; Yamashita, Y.; Onaka, K.; Sakurai, Y.; Kondo, N.; Ono, K.; Uto, Y.; Hori, H. Design, synthesis and destructive dynamic effects of BODIPY-containing and curcuminoid boron tracedrugs for neutron dynamic therapy. Anticancer Res. 2011, 31, 2477-2481.
